# Accuracy of the Triple Test Versus Colposcopy for the Diagnosis of Premalignant and Malignant Cervical Lesions

**DOI:** 10.31557/APJCP.2020.21.12.3501

**Published:** 2020-12

**Authors:** Neda Fattahi Meybodi, Mojgan Karimi-Zarchi, Leila Allahqoli, Leila Sekhavat, Georgios Gitas, Azam Rahmani, Arezoo Fallahi, Babak Hassanlouei, Ibrahim Alkatout

**Affiliations:** 1 *Shahid Sadoughi University of Medical Science, Yazd, Iran. *; 2 *Endometriosis Research Center, Iran University of Medical Sciences (IUMS), Tehran, Iran. *; 3 *Department of Obstetrics and Gynecology, University Hospital Schleswig Holstein, Luebeck, Germany. *; 4 *Nursing and Midwifery Care Research Center, School of Nursing and Midwifery, Tehran University of Medical Sciences, Tehran, Iran. *; 5 *Social Determinants of Health Research Center, Research Institute for Health Development, Kurdistan University of Medical Sciences, Sanandaj, Iran. *; 6 *Dpartment of Epidemiology, School of Public Health, Iran University of Medical Sciences, (IUMS), Tehran, Iran. *; 7 *University Hospitals Schleswig-Holstein, Campus Kiel, Kiel School of Gynaecological Endoscopy. Arnold-Heller-Str. 3, Haus 24, 24105 Kiel, Germany. *

**Keywords:** Diagnostic accuracy-, Pap smear, VIA- VILI, colposcopy, cervical lesions

## Abstract

**Background:**

Despite the World Health Organization (WHO) recommendations concerning the use of alternative tests for the detection of cervical cancer precursor lesions in low-income countries, the accuracy of these tests is a debated issue. In the present study we compare the diagnostic accuracy of the triple test with that of colposcopy for the diagnosis of premalignant and malignant cervical lesions.

**Methods:**

A cross-sectional study was performed in 328 women referred to the gynecology clinic at Shahid Sadoughi Hospital, affiliated to Yazd University of Medical Sciences (SSUMS), Yazd, Iran, from March 2016 to June 2018. As the first step, a Pap smear was obtained from all participants. Visual inspection with acetic acid (VIA) and Lugol’s iodine (VILI) was performed in accordance with the known protocol. A colposcopy was then conducted in all participants, biopsy samples were obtained, and histological features studied. Finally, the results were compared by statistical analysis.

**Results:**

The age range of the participants was 30 - 50 years. Of 328 women, 60 (18.3 %) were postmenopausal. Two-hundred and five patients (62.5 %) had an abnormal Pap smear, 165 (50.3 %) had abnormal results on colposcopy, and 141 (43 %) had abnormal histopathology reports. The VIA was positive in 129 patients (39.3 %) and the VILI in 177 (54 %). The results of the triple test were reported to be positive in 205 cases (51.52 %). The sensitivity of the triple test in the detection of premalignant and malignant cervical lesions was 78.7 % and 69 %, respectively. The sensitivity and specificity of colposcopy in the detection of premalignant and malignant cervical lesions was 80.1 % and 72.2 %, respectively. The diagnostic accuracy of the triple test and colposcopy in the detection of premalignant and malignant cervical lesions was 73 % versus 75 %.

**Conclusion:**

Since the results of the study showed that the diagnostic accuracy of the triple test is equivalent that of colposcopy, the former may be used in low-income countries and areas lacking access to colposcopy.

## Introduction

Cervical cancer is the fourth most common type of cancer in women worldwide (Consul et al., 2012). Due to the absence of effective and regular screening programs aimed at identifying and treating precancerous lesions, and the absence of human papillomavirus (HPV) vaccines (Karimi-Zarchi et al., 2020), cervical cancer is the most common gynecologic cancer in some developing countries (Consul et al., 2012, Bray et al., 2018). The disease is considered preventable because of its long period of premalignancy and organized screening was shown to reduce morbidity and mortality rates (Pradhan et al., 2007, Barut et al., 2015). The management of cervical cancer should be personalized taking into account the performance status of the patient (Vitale et al., 2019), and stage of cancer (Casarin et al., 2020). 

The gold standard for the detection of cervical lesions is a biopsy obtained by colposcopy, followed by histological analysis (IARC, 2011). While this strategy is very successful in high-income countries, its implementation requires a well-organized, complex, and expensive infrastructure, as well as trained personnel, including cytopathologists, colposcopy specialists, and pathologists (Rambau, 2011, Catarino et al., 2018). 

The World Health Organization (WHO) recommends alternative tests, including visual inspection of the cervix after application of 3–5 % acetic acid (VIA) or Lugol’s iodine (VILI) for the detection of cervical cancer precursors in low-income countries (WHO and IARC, 2012, Huy et al., 2018, Catarino et al., 2018). Recently, VIA and VILI tests were used along with the Pap smear to improve screening results (Catarino et al., 2018, Huy et al., 2018). These tests are considered affordable and particularly suitable for the detection of premalignant and malignant cervical lesions in low-income countries. The results of the tests are obtained immediately, thus permitting same-day screening and treatment (Goldie et al., 2005, Sankaranarayanan et al., 1998). Despite WHO recommendations for the use of these tests in low-income countries (Consul et al., 2012, WHO and IARC, 2012), their accuracy has been questioned in some studies (Bhattacharyya et al., 2015, Catarino et al., 2018). We aimed to investigate the accuracy of the triple test and colposcopy for the diagnosis of premalignant and malignant cervical lesions. 

## Materials and Methods

*Study design and patients*

A cross-sectional study was conducted in women who were referred to the gynecology clinic at Shahid Sadoughi Hospital affiliated to Yazd University of Medical Sciences (SSUMS), Yazd, Iran, from March 2016 to June 2018. The aim of the investigation was to compare the accuracy of the triple test with that of colposcopy for the diagnosis of premalignant and malignant cervical lesions. The triple test consisted of a combination of the Pap smear, VIA, and VILI test. 

Based on similar investigations in the past, we determined a sample size of 325 patients (α= 0.05, power: 0.09) (Consul et al., 2012). All married women who were referred to the gynecology clinic and were candidates for colposcopy in accordance with American Society for Colposcopy and Cervical Pathology (ASCCP) guidelines (Wentzensen et al., 2017) were enrolled in the study. Women with a history of endometrial, ovarian or cervical cancer, a history of recent cervical manipulation (previous conization, cryotherapy, etc.), or an abnormal Pap smear were not eligible for the study. Women who became pregnant were unwilling to cooperate, or were not referred for colposcopy/biopsy to our center were also excluded from the investigation. 

Patients were informed about the study and written consent was obtained from all of the participants. Gynecological examinations, including investigations of the breast and pelvis, were performed. General parameters including age, parity, menopausal status, mode of delivery, and the indication for colposcopy were either obtained directly from the patients or extracted from their medical records. 

Each patient was placed in the dorsal lithotomy position. A speculum was placed in the patient’s vagina. The cervix was examined with the naked eye and with a speculum. A smear was taken with a cytobrush and evaluated by the Bethesda system (Soloman, 1989). Then, for visual inspection with VIA, the cervix was washed with a sterile saline solution and 4% acetic acid was applied with a cotton swab. The cervix was examined after one minute and the results were evaluated on the basis of diagnostic criteria for VIA (Sankaranarayanan et al., 2003). 

Subsequently, VILI was applied on the cervix with a cotton swab according to the instructions (Sankaranarayanan and Wesley, 2003) and the results were recorded after one minute, based on the diagnostic criteria for VILI (Sankaranarayanan et al., 2003). 

Each patient was then referred for colposcopy and biopsy, and lesions were graded on the basis of Reid’s colposcopic index (Ferris and Greenberg, 1994). Biopsies were taken from various areas using separate instruments, and histological features were studied. The specimens were then stained with hematoxylin and eosin, and checked by a clinical pathologist blinded to the Pap smear results. VIA, VILI, Pap smear tests, and colposcopy were performed by a gynecological oncologist who was completely unaware of the purpose of the study. All Pap testing and histologic evaluations were performed in the same laboratory. The results of the Pap smear, VIA, VILI, visual inspection of the cervix, and histological biopsy were recorded on the checklist and the patients’ medical records. A Pap smear test result of atypical squamous cells of undetermined significance (ASCUS) or worse was considered a positive test. The results of the VIA and VILI tests were interpreted according to the respective criteria (Sankaranarayanan et al., 2003). The result of the triple test was considered positive when at least two or more tests (Pap smear, VIA, and VILI) were positive; in all other cases, the triple test was deemed negative. On histological investigation, a cervical intraepithelial neoplasia (CIN) lesions 1 or worse was considered positive. 

*Statistical analysis*

Commercially available software (IBM SPSS Statistics version 22; IBM, Chicago, IL, USA) was used for data analysis. Descriptive statistics (frequency and percent) were used to present the data. Diagnostic accuracy measures show the ability of a test to discriminate between and/or predict disease and health. This discriminative ability is quantified by several measures of diagnostic accuracy: sensitivity and specificity; positive and negative predictive values (PPV, NPV); the area under the receiver operating characteristic (ROC) curve (AUC); and the overall diagnostic accuracy (Eusebi, 2013). In the present study, diagnostic accuracy measures included the sensitivity, specificity, PPV, NPV, and the overall diagnostic accuracy of the triple test and colposcopy. The usefulness of the test was demonstrated by the ROC-AUC. The ROC curve of the triple test in plotting sensitivity versus 1− speciﬁcity was computed. P values <0.05 or less were considered statistically significant. 

## Results

**Figure 1:**
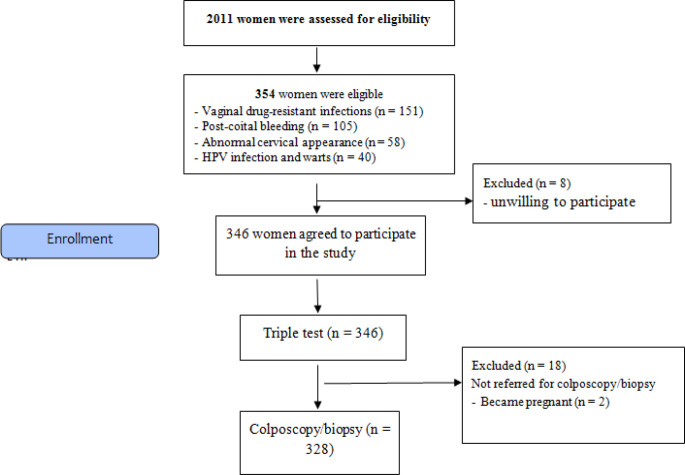
Diagram of the Study

Of 2011 women referred to the gynecology clinic, 354 were eligible for the study. Of these, 346 women agreed to participate in the study. The indication for colposcopy was as follows: vaginal drug-resistant infections (n = 151, 42.65 %), post-coital bleeding (n = 105, 29.66 %), abnormal appearance of the cervix (n = 58, 16.38 %), HPV infection and warts (n = 40, 11.3 %). Twenty-six patients (7.34 %) were excluded due to pregnancy, unwillingness to continue their participation, or because they had not been referred for colposcopy/biopsy. Ultimately, the data of 328 patients were available for analysis (Figure 1). The most frequent age group was 30-50 years; the patients’ mean age was 41 ± 5.15 years. Of 328 participants, 60 (18.3 %) were postmenopausal. The majority of the women (58 %) had had two vaginal deliveries. 

The results of the triple test, colposcopy, and biopsy were as follows: 205 patients (62.5 %) had an abnormal Pap smear, 165 (50.3 %) had abnormal colposcopic findings, and 141 (43 %) had abnormal histopathology reports. The VIA test was positive in 129 patients (39.3 %), and the VILI test in 177 (54 %). The results of the triple test (separately), colposcopy, and biopsy are summarized in Table 1.

Based on the data shown in Table 1, ASCUS was the most abnormal Pap smear result (118 of 205 patients; 36 %). CIN 1 was the most abnormal finding on colposcopy (120 of 165 patients; 36.6 %) and biopsy (104 of 187 patients; 31.4 %). The distribution frequency of the results of the Pap smear, colposcopy, and biopsy are presented in Table 1. The triple test was positive in 169 of 328 patients (51.52 %). According to the data listed in Table 2, the results of the triple test and histopathology were consistent in 240 of 328 patients (73.17 %). Likewise, the results of colposcopy were consistent with pathological reports in 248 of 328 patients (75.61 %). Sensitivity, specificity, PPV, NPV were determined for the triple test and colposcopy; the results are summarized in Table 2. The sensitivity and specificity of the triple test in the detection of premalignant and malignant cervical lesions were reported to be 78.7 % and 69 %, respectively. The sensitivity and specificity of colposcopy in the detection of premalignant and malignant cervical lesions were reported to be 80.1 % and 72.2 %, respectively (Table 2).

The sensitivity, specificity, NPV, and PPV of the triple test and colposcopy were similar in regard of the detection of premalignant cervical lesions (Table 3). The specificity and NPV of colposcopy were higher than those of the triple test in the detection of malignant cervical lesions. The diagnostic accuracy indicators of the triple test and colposcopy for the detection of premalignant and malignant cervical lesions are shown in Table 3. 

The diagnostic accuracy of the triple test and colposcopy in the detection of premalignant cervical lesions was reported to be 75 % and 73 %, respectively. The diagnostic accuracy of the triple test and colposcopy in the detection of malignant cervical lesions was 58 % and 94 %, respectively (Table 3).

A ROC analysis was performed to compare the diagnostic value of the triple test and colposcopy in the detection of premalignant and malignant cervical lesions. As shown in Table 4, the diagnostic performance of the triple test and colposcopy were similar: AUC [(95 % conﬁdence interval (CI)] was 0.64 (95 % CI; 0.58 - 0.70) vs. 0.65 (95 % CI; 0.59 - 0.71). The difference in ROC-AUC (95 % CI) between the triple test and colposcopy was not significant (Table 4).

**Figure 2:**
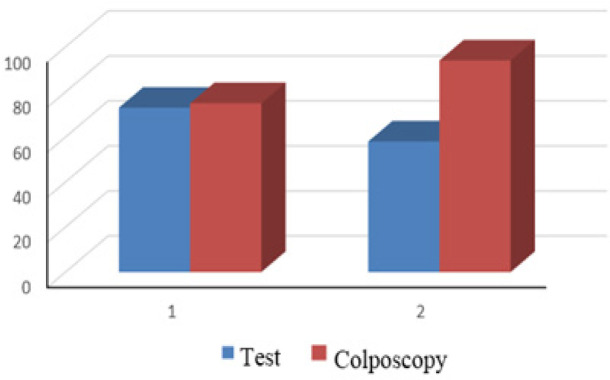
Diagnostic Accuracy of the Triple Test and Colposcopy in the Detection of Premalignant and Malignant Cervical Lesions. (1. Premalignant cervical lesions 2. Malignant (CIN II and worse) cervical lesions

**Figure 3:**
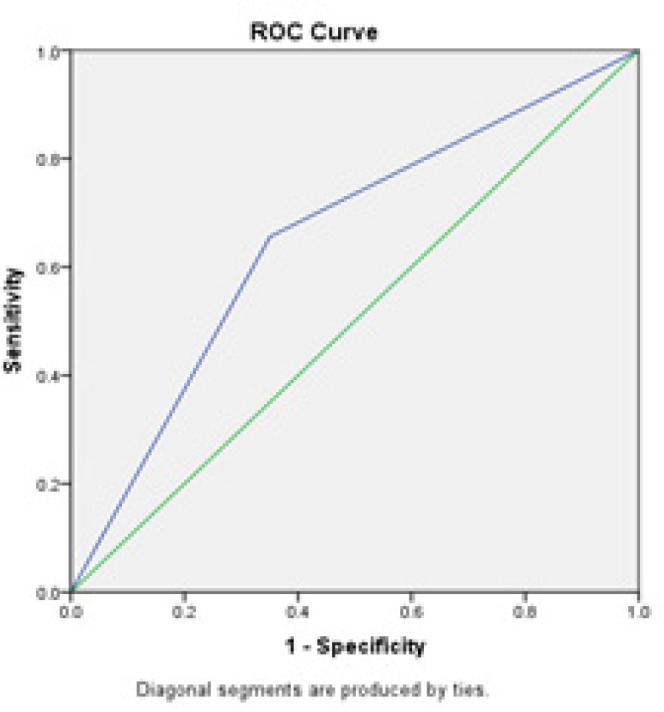
ROC Curve of Colposcopy for Premalignant and Malignant Cervical Lesions. The diagonal divides the ROC space. Points above the diagonal express a classification better than random

ROC curves of the triple test and colposcopy in the detection of premalignant and malignant cervical lesions are shown in Figures 2 and 3. 

**Table 1:** Distribution Frequency of the results of the Triple Test, Colposcopy, and Biopsy

Test	Result	Frequency (%)	Result	Frequency (%)
Pap smear	Normal	123 (37.5)		
	Abnormal	205 (62.5)	ASCUS	118 (36)
			HSIL	26 (7.9)
			LSIL	38 (11.6)
			ASC-H	21 (6.4)
			AGUS	2 (0.6)
VIA	Negative	199 (60.7)		
	Positive	129 (39.3)		
VILI	Negative	151 (46)		
	Positive	177 (54)		
Colposcopy	Normal	163 (49.7)		
	Abnormal	165 (50.3)	CIN 1	120 (36.6)
			CIN 2	32 (9.8)
			CIN 3	13 (4)
Biopsy histology	Normal	187 (57)		
	Abnormal	141 (43)	CIN 1	104 (31.7)
			CIN 2	20 (6.1)
			CIN 3	9 (2.7)
			AIS	8 (2.4)

Abbreviations: VIA, Visual inspection with acetic acid; VILA, Visual inspection with Lugol's iodine; HSIL, High-grade squamous intraepithelial lesion; LSIL, Low-grade squamous intraepithelial lesion; ASCUS, Atypical squamous cells of undetermined significance; ASC-H, Atypical squamous cells, cannot exclude HSIL; AGUS, Atypical glandular cells of undetermined significance; CIN, Cervical intraepithelial neoplasia

**Table 2:** Results of the Triple Test and Colposcopy Compared with Biopsy in the Detection of Premalignant and Malignant Cervical Lesions

Test		Biopsy†		Total
		Positive	Negative	
		Frequency (%**)		
Triple test*	Positive	111 (78.7**)	58	169
	Negative	30	129 (69**)	159
Colposcopy	Positive	113 (80.1**)	52	165
	Negative	28	135 (72.2**)	163
Total		141	187	328

* The triple test included the Pap smear, VIA, and VILI test; **Percentages were calculated on the basis of sensitivity and specificity formulas; †CIN 1 and more was considered a positive result.

**Table 3:** Diagnostic Accuracy Indicators of the Triple Test and Colposcopy in Regard of Premalignant and Malignant Cervical Lesions

Index	CIN 1 lesion and worse		CIN 2 lesions and worse	
	Triple test (95% CI)	Colposcopy (95% CI)	Triple test (95% CI)	Colposcopy (95% CI)
Sensitivity	78.7 (74.3 - 83.2)	80.1 (75.5 - 84.4)	91.9 (88.9 - 94.8)	86.5 (82.8 - 90.2)
Specificity	69 (65 - 74.9)	72.2 (67.3 - 77)	53.6 (48.2 - 58.9)	95.5 (93.2 - 97.7)
NPV	81.1 (76.8 - 85.3)	82.8 (78.7 - 86.8)	98 (96.5 - 99.5)	98.2 (96.7 - 99.6)
PPV	65.68 (63.4 - 73.5)	65.7 (60.5 - 70.8)	20 (15.6 - 24.3)	71.1 (66.2 - 76)
Diagnostic accuracy	75 (70.3 - 79.7)	73 (68.2 - 77.8)	58 (52.6 - 63.3)	94 (91.4 - 96.6)

Abbreviations: CI, confidence interval; NPV, negative predictive value; PPV, positive predictive value; The triple test consisted of the Pap smear, VIA, and the VILI test

**Figure 4:**
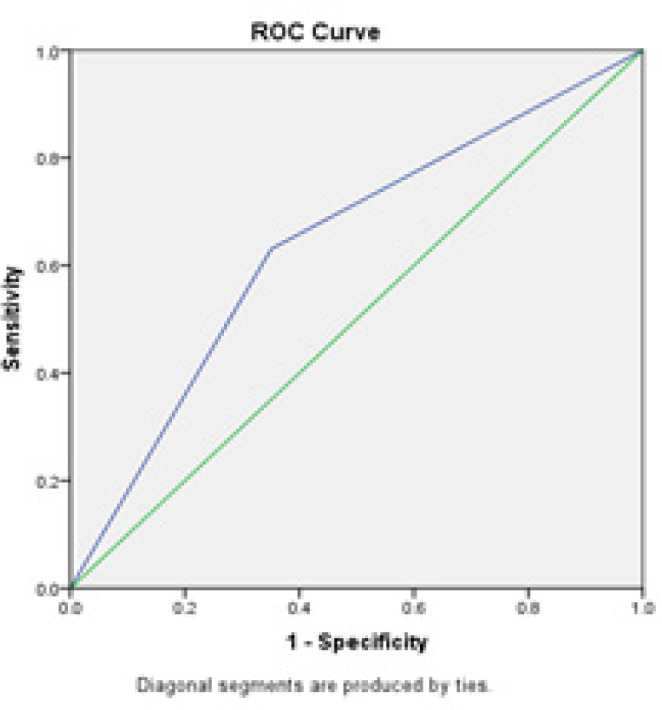
ROC Curve of Colposcopy for Premalignant and Malignant Cervical Lesions

**Table 4:** ROC-AUCs Curves of the Triple Test and Colposcopy in the Diagnosis of Premalignant and Malignant Cervical Lesions

		ROC-AUC (95% CI)	
Biopsy	N (%)	Triple test*	Colposcopy
Positive	187 (57)		
Negative	141 (43)	0.64 (0.580 - 0.700)	0.653 (0.593 - 0.712)
p-value	0.35		

* The triple test included the Pap smear, VIA, and the VILI test

## Discussion

Given the high incidence of cervical cancer and the importance of prompt diagnosis and treatment, scientists have given substantial attention to the development of accurate and cost-effective methods for screening and diagnosing this type of cancer (Barut et al., 2015, Huy et al., 2018, Valenti et al., 2017, Rossetti et al., 2017). The present study aimed to compare the diagnostic accuracy of the triple test and colposcopy for the diagnosis of premalignant and malignant cervical lesions. The diagnostic accuracy of the triple test and colposcopy was similar with regard to the detection of premalignant cervical lesions. The diagnostic accuracy of the triple test and colposcopy in the detection of premalignant cervical lesions was reported to be 75 % and 73 %, respectively. The diagnostic accuracy of colposcopy in the detection of malignant cervical lesions was superior to that of the triple test. The AUC was similar for the triple test and colposcopy. 

Various studies have addressed the accuracy of tests for the diagnosis of cervical cancer and reported contradictory results (Barut et al., 2015, Bhattacharyya et al., 2015, Davies et al., 2015, Dawood and El-Tahmoudy, 2015, Catarino et al., 2018, Huy et al., 2018, Rossetti et al., 2017, Valenti et al., 2017). 

In the present study, the sensitivity and specificity of the triple test and colposcopy were within the ranges reported in other studies (Consul et al., 2012, Sankaranarayanan et al., 2003, Dawood and El-Tahmoudy, 2015, Barut et al., 2015, Davies et al., 2015, Karimi-Zarchi et al., 2013). The sensitivity and specificity of combined use of the Pap smear, VIA, and VILI were higher than that of the individual tests. The combined test was equivalent to colposcopy in the detection of premalignant cervical lesions. Previous studies have also shown that combinations of tests are associated with a higher sensitivity and specificity for the diagnosis of premalignant and malignant cervical lesions (Consul et al., 2012, Sankaranarayanan et al., 2003, Dawood and El-Tahmoudy, 2015). In Sarian and co-workers’ study, VIA and VILI used as stand-alone tests failed to detect malignant cervical cancer, whereas the combined use of VIA and VILI markedly improved their performance as screening tools for the detection of malignant cervical lesions (Sarian et al., 2005). 

In the present study, the PPV and NPV of the triple test and colposcopy were the same for the detection of premalignant and malignant cervical lesions. A large majority of scientists regard PPV and NPV as the main parameters of a screening test. However, it should be noted that PPV and NPV depend on the population being tested and the technical characteristics of the screening test (Goetzinger and Odibo, 2011, Šimundić, 2009, Maxim et al., 2014). 

In the present study, the PPV of the triple test was lower and its NPV higher compared to colposcopy for the detection of malignant cervical lesions (CIN 2 or worse). Indeed, applying the triple test for the detection of malignant cervical lesions (CIN 2 or worse) yielded more numerous false positives cases compared to colposcopy. This finding was in line with the data published by Counsel and co-workers (Consul et al., 2012). A desirable screening test should have a high PPV and a low NPV (Goetzinger and Odibo, 2011). In contrast, we registered a high NPV and a low PPV. Since this would involve higher costs, the value of the test should be investigated further. It should be noted that the predictive value of a test is highly dependent on the prevalence of the disease in the examined population (Goetzinger and Odibo, 2011, Maxim et al., 2014). Therefore, the predictive value of a test cannot be directly compared between studies(Maxim et al., 2014).

As diagnostic accuracy is one criterion to measure the effectiveness of a test (Eusebi, 2013), we determined diagnostic accuracy according to the number of correctly classified persons (TP + TN) among all subjects (TP + TN + FP + FN). The diagnostic accuracy of the triple test and colposcopy in the detection of premalignant and malignant cervical lesions was approximately the same. In fact, the findings of the present study confirm the WHO recommendations in regard of using alternative methods such as the triple test for cervical cancer screening in low-income countries lacking access to colposcopy (WHO and IARC, 2012). In the present study, although the diagnostic accuracy of the triple test and colposcopy was similar for the diagnosis of premalignant cervical lesions, the accuracy of the triple test for the detection malignant cervical lesions (CIN 2 or worse) was inferior to that of colposcopy (58 % vs. 94 %). This finding is consistent with the data reported in other studies (Consul et al., 2012). 

We performed a ROC analysis to compare the performance of the triple test and colposcopy in the detection of premalignant and malignant cervical lesions. The ROC-AUC shows the performance of the test in distinguishing health from disease or any other two conditions of interest (Maxim et al., 2014, Šimundić, 2009). In the present investigation, the triple test and colposcopy had a similar AUC (0.64 vs. 0.65). Any AUC value between 0 and 1 is reported to be a good indicator of the accuracy of a test (Šimundić, 2009, Maxim et al., 2014). Accordingly, both methods possessed good diagnostic accuracy. On the other hand, the difference between the triple test and colposcopy in regard of the AUC was not significant. Given the equal performance of the triple test and colposcopy for the detection of premalignant and malignant cervical lesions, the triple test may be recommended for the diagnosis of cervical lesions in areas lacking access to colposcopy due to costs, infrastructure, or the absence of suitable specialists.

In summary, with late diagnosis and treatment the number of patients who face the long-term consequences of cervical cancer increases (Laganà et al., 2019, Laganà et al., 2017). So the diagnosis of cervical cancer is essential. The present study showed that the diagnostic accuracy of the combination of the Pap smear, VIA, and VILI was similar to that of colposcopy in the detection of premalignant and malignant cervical lesions. Thus, this combined test may serve as a suitable alternative screening test in low-income countries with no access to colposcopy. Although we conducted a thorough survey and all tests were performed by a gynecological pathologist, the study was limited by the small sample size. The present results need to be proven in larger populations with the same prevalence of disease. 

In conclusion, based on the results of the present study, the triple test may be suggested for the diagnosis of premalignant and malignant and cervical lesions. Since it is simple to use, cost effective, accessible, and can be taught easily, the triple test may be regarded as an appropriate alternative to colposcopy in low-income countries. 

*Abbreviations*

ASCUS: Atypical squamous cells of undetermined significance

ASC-H: Atypical squamous cells, cannot exclude HSIL

AGUS: Atypical glandular cells of undetermined significance

CIN: Cervical intraepithelial neoplasia

HSIL: High-grade squamous intraepithelial lesion

LSIL: Low-grade squamous intraepithelial lesion 

VIA: Visual inspection with acetic acid

VILA: Visual inspection with Lugol’s iodine

## Data Availability

The data sets generated and analyzed are available from the corresponding author under reasonable request and with permission from Shahid Sadoughi University of Medical Sciences (SSUMS), Yazd, Iran.
